# P-1931. Aspergillosis-Attributable Death in the United States: Analysis of Death Certificate Data

**DOI:** 10.1093/ofid/ofaf695.2099

**Published:** 2026-01-11

**Authors:** Craig I Coleman, Dakota Sicignano, Matthew Mastropietro, Belinda Lovelace, Thomas J Walsh

**Affiliations:** University of Connecticut School of Pharmacy, Storrs, CT; University of Connecticut School of Pharmacy, Storrs, CT; University of Connecticut School of Pharmacy, Storrs, CT; F2G, Inc., Princeton, New Jersey; Center for Innovative Therapeutics and Diagnostics (citdx.org), Richmond, VA

## Abstract

**Background:**

Studies have evaluated mycoses-related death certificates but data on aspergillosis-attributable death is sparse. We characterized death certificates listing aspergillosis as a cause of death across demographic and comorbidity strata.

**Figure:**
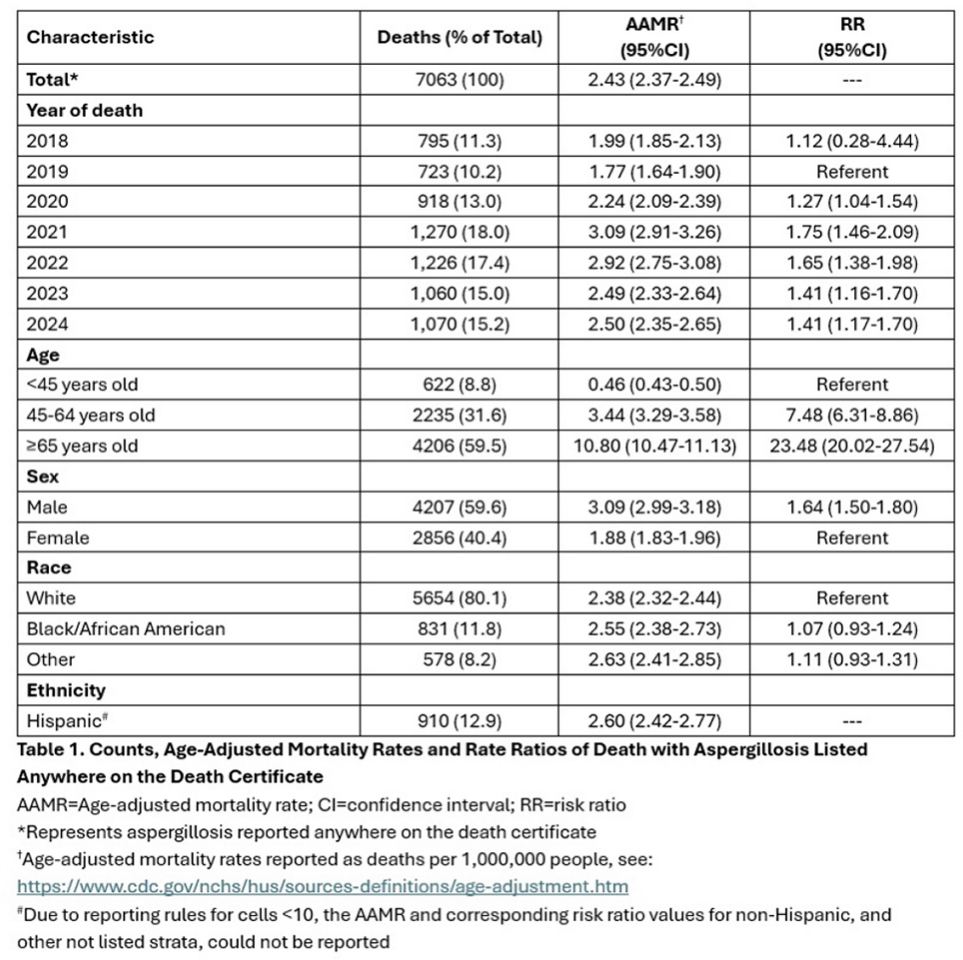

**Figure:**
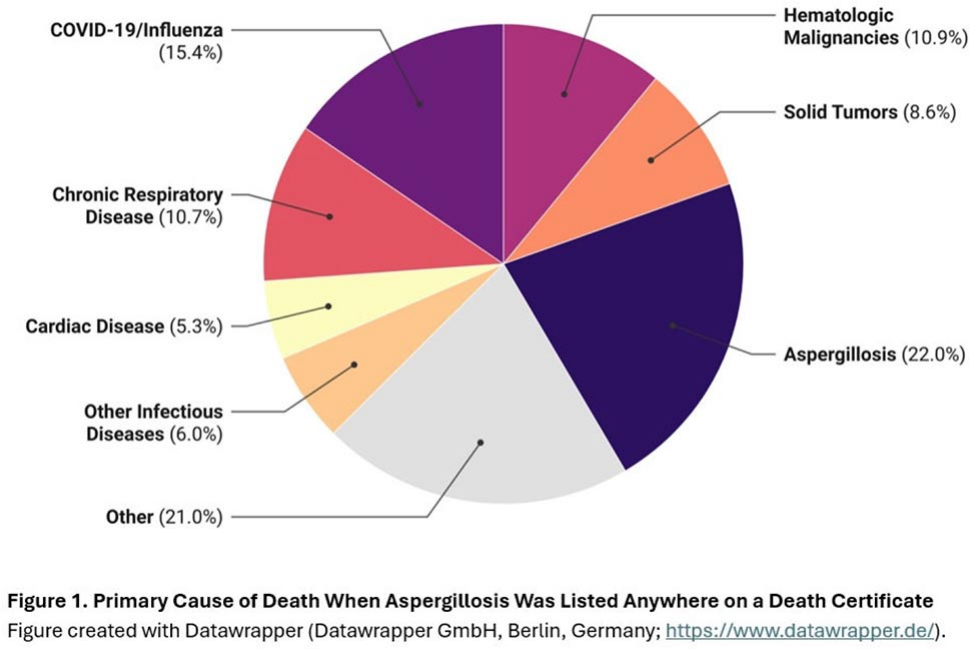

**Methods:**

We analyzed data from the United States National Vital Statistics System for 2018-2024. Aspergillosis-attributable death was identified by the presence of ≥1 International Classification of Diseases-10^th^ Revision diagnosis code of B44.x listed anywhere on a death certificate. We present age-adjusted mortality rates (AAMRs)/1,000,000 people with 95% confidence intervals (CI) for aspergillosis deaths and stratified them by demographics. Risk ratios (RR) were used to compare AAMRs across strata and the proportion of aspergillosis vs. non-aspergillosis death certificates listing key comorbidities.

**Figure:**
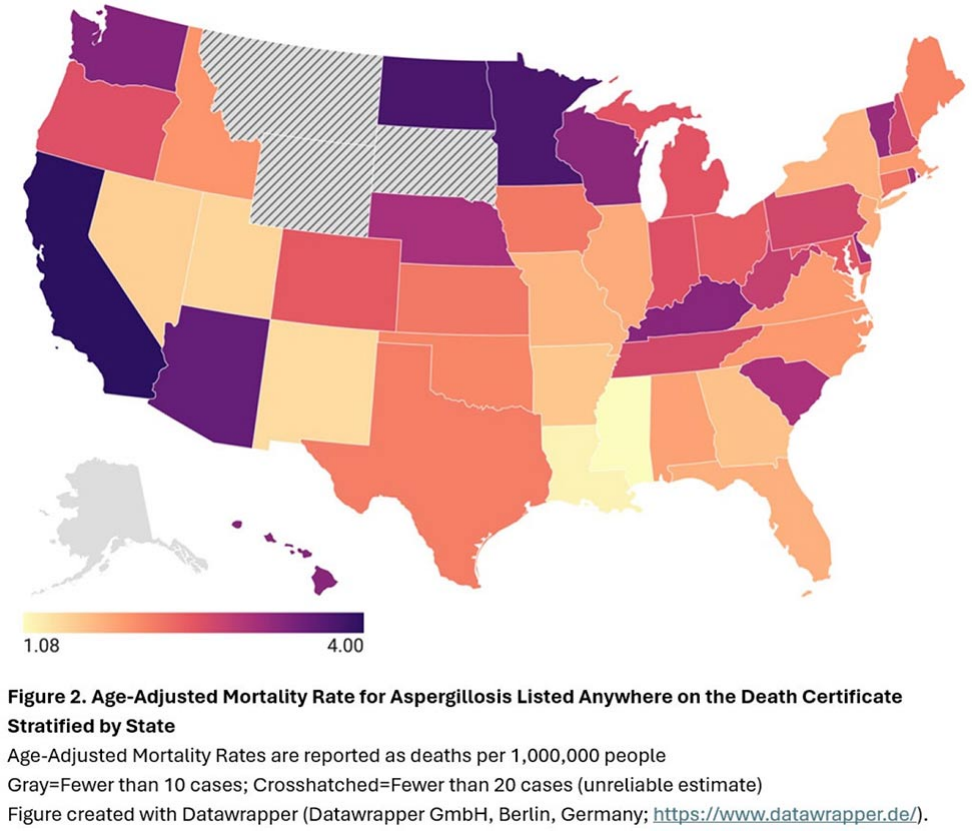

**Figure:**
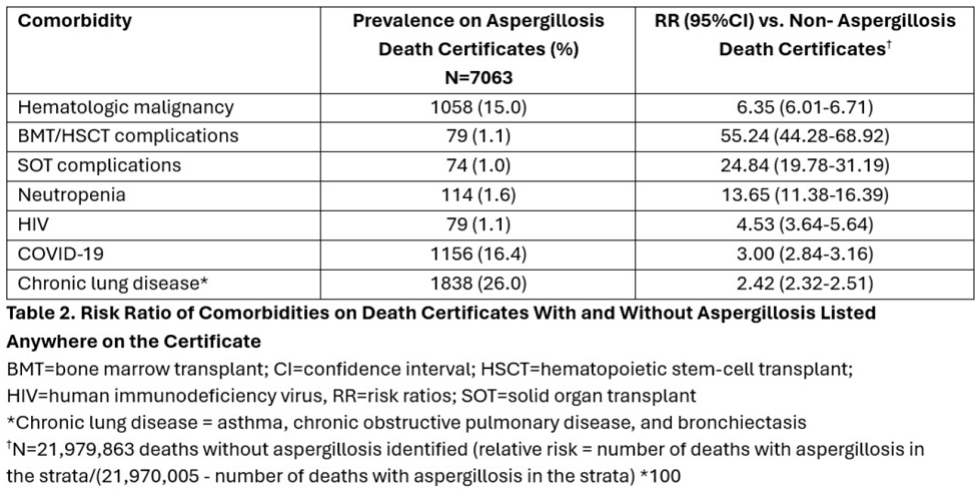

**Results:**

There were 7063 aspergillosis deaths (AAMR: 2.43, 95%CI 2.37-2.49/1,000,000) (Table 1). Of these, 1551 (22.0%) death certificates had aspergillosis listed as the underlying (primary) cause (AAMR: 0.53, 95%CI 0.54-0.56) (Figure 1). Codes for “invasive aspergillosis disease” (B44.0, B44.1, B44.7) were most frequent (n=4880, 69.1%); specifically, the code for “other pulmonary aspergillosis” (B44.1, n=4292, 60.7%). Unspecified aspergillosis (B44.9) was listed on 2139 certificates. Nearly 60% of aspergillosis deaths occurred in ≥65-year-olds (RR: 23.48, 95%CI 20.02-27.54), and 59.6% in males vs. females (RR: 1.64, 95%CI 1.50-1.80). Race and ethnicity were not associated with differences in AAMR. AAMRs varied widely across states (Figure 2). Hematologic malignancy, hematopoietic cell and solid organ transplant, neutropenia, human immunodeficiency virus, COVID-19 and chronic lung disease were more likely present on death certificates of those with vs. without aspergillosis (Table 2).

**Conclusion:**

The AAMR for aspergillosis was 2.43 (95%CI 2.37-2.49)/1,000,000, higher than previously reported rates. Sex and geography impacted AAMRs. Despite available antifungals, AAMRs have not declined. Most certificates listed “other pulmonary aspergillosis” and those with hematologic malignancies, COVID-19 and chronic lung disease were at highest risk.

**Disclosures:**

Craig I. Coleman, PharmD, F2G Inc.: Advisor/Consultant|F2G Inc.: Grant/Research Support Belinda Lovelace, PharmD, MS, MJ, F2G Inc.: Employee Thomas J. Walsh, MD, PhD (Hon), FIDSA, FAAM, FECMM, Allergan: Grant/Research Support|Astellas: Advisor/Consultant|Astellas: Grant/Research Support|Basilea: Advisor/Consultant|F2G Inc.: Advisor/Consultant|F2G Inc.: Grant/Research Support|Gilead: Advisor/Consultant|Gilead: Grant/Research Support|Karyopharm: Advisor/Consultant|Lediant: Advisor/Consultant|Lediant: Grant/Research Support|Merck: Advisor/Consultant|Merck: Grant/Research Support|Partner Therapeutics: Advisor/Consultant|Scynexis: Advisor/Consultant|Scynexis: Grant/Research Support|Shionogi: Advisor/Consultant|Shionogi: Grant/Research Support|Statera: Advisor/Consultant|T2 Biosystems: Advisor/Consultant|T2 Biosystems: Grant/Research Support|Viosera: Grant/Research Support

